# Measures of Fine Motor Skills in People with Tremor Disorders: Appraisal and Interpretation

**DOI:** 10.3389/fneur.2013.00050

**Published:** 2013-05-10

**Authors:** Kathleen E. Norman, Martin E. Héroux

**Affiliations:** ^1^School of Rehabilitation Therapy, Queen’s UniversityKingston, ON, Canada; ^2^Centre for Neuroscience Studies, Queen’s UniversityKingston, ON, Canada; ^3^Neuroscience Research AustraliaSydney, NSW, Australia

**Keywords:** movement disorders, tremor, fine motor skills, dexterity, outcome measures, measurement

## Abstract

People with Parkinson’s disease, essential tremor, or other movement disorders involving tremor have changes in fine motor skills that are among the hallmarks of these diseases. Numerous measurement tools have been created and other methods devised to measure such changes in fine motor skills. Measurement tools may focus on specific features – e.g., motor skills or dexterity, slowness in movement execution associated with parkinsonian bradykinesia, or magnitude of tremor. Less obviously, some tools may be better suited than others for specific goals such as detecting subtle dysfunction early in disease, revealing aspects of brain function affected by disease, or tracking changes expected from treatment or disease progression. The purpose of this review is to describe and appraise selected measurement tools of fine motor skills appropriate for people with tremor disorders. In this context, we consider the tools’ content – i.e., what movement features they focus on. In addition, we consider how measurement tools of fine motor skills relate to measures of a person’s disease state or a person’s function. These considerations affect how one should select and interpret the results of these tools in laboratory and clinical contexts.

A reduction in upper extremity function and fine motor skills is a common consequence of living with a tremor disorder (Feys et al., 2002; Héroux et al., 2006; Dibble et al., 2010). Hand tremor, even when mild, causes difficulties with everyday tasks such as writing, self-care, and fine object manipulation. These activity limitations can lead to participation restrictions, social isolation, and a reduced quality of life (Diamond and Jankovic, 2005; Benito-Leon and Louis, 2006; Lorenz et al., 2006). Such consequences are made worse by embarrassment that often accompanies tremor (Bain et al., 1994; Louis and Rios, 2009). Although measuring tremor severity is important in clinical and research settings, it is just as important to measure the impact of tremor on fine motor skills and upper extremity function. Appropriate measurement of these constructs will ultimately influence patient care, intervention, and service prescription, as well as policy, and funding decisions (Hobart, 2003; Hobart et al., 2007). In the context of tremor disorders, clinicians, and researchers do not always measure activity limitations (Gorman et al., 1986; Sasso et al., 1991; Obwegeser et al., 2001; Vaillancourt et al., 2003; Fox et al., 2004; Van Der Walt et al., 2012), or they use tools with poor, unknown, or inadequate measurement properties for the targeted population (Herzog et al., 2003; Flora et al., 2010; Thevathasan et al., 2011; Ohye et al., 2012; Zappia et al., 2013).

The purpose of this mini-review is to (1) describe why clinicians and researchers should include measures of upper extremity function and fine motor skills in their evaluation of individuals with tremor disorders, (2) provide an overview of key measurement properties and attributes to be considered when selecting measurement tools, and (3) appraise and interpret several measures of fine motor skills that have been used in people with tremor disorders and consider other measures that may be useful. This mini-review does not represent an exhaustive review of available measurement tools. Rather, it is intended to focus on how measuring fine motor skills in people with tremor disorders can benefit from considering the wider literature about the development of scientifically sound clinical measures in general and hand function evaluation in particular.

## Tremor Disorders and Activity limitations

Movement disorders are a group of diseases and syndromes affecting a person’s ability to produce and control movement, with tremor disorders being the most common (Deuschl et al., 1998). Most affected are the upper extremities, for example, the resting tremor of Parkinson’s disease (PD), the action tremor that characterizes essential tremor (ET), or the intention tremor that develops following cerebellar damage [for a review see Deuschl et al. (2001)]. Functionally, tremor impacts the performance of fine motor skills such as feeding, drinking, writing, body care, and fine object manipulation (Feys et al., 2002; Héroux et al., 2006; Dibble et al., 2010), and results in activity limitation in 50–75% of those living with upper extremity tremor from ET (Koller et al., 1986; Bain et al., 1994; Dogu et al., 2005).

An important part of evaluating individuals with tremor disorders is determining the type (e.g., rest, postural, kinetic, intention) and severity of tremor. Clinically this involves observing patients holding various postures and executing specified movements and assigning an ordinal scale rating. The use of accelerometers, digitizing tablets, and other technology can enable more precise measures of tremor severity (Elble et al., 1996, 2006; Norman et al., 2011). Clinical trials investigating the efficacy of pharmacological agents and neurosurgical techniques often focus on measures of tremor severity as their primary (or sole) outcome measure, especially in ET (e.g., Sasso et al., 1991; Obwegeser et al., 2001; Vaillancourt et al., 2003). The logic of this approach is evident: the intervention aims to reduce tremor amplitude. However, the ultimate therapeutic aim is to induce clinically meaningful improvements in functional performance and it should not be assumed that a reduction in tremor amplitude will result in a meaningful improvement in fine motor skills. Several studies have found weak or absent correlations between tremor amplitude and upper extremity function (Bain et al., 1993; Louis et al., 1999, 2001; Héroux et al., 2006), although this appears to depend on how tremor is measured (Norman et al., 2011). The lack of a strong relationship between a person’s level of impairment and disability has been noted in other neurological and non-neurological conditions (O’Neill et al., 1998; Ploutz-Snyder et al., 2002; Hoang et al., 2012; Carvalho et al., 2013), and highlights the need to include measures of activity limitations.

In short, there is often a need to evaluate upper extremity function and fine motor skills in individuals with tremor disorders. Selecting an appropriate measurement tool is essential and should be founded on a clear understanding of the scientific quality of potential tools and their usefulness in the clinical setting (Finch et al., 2002; Hobart, 2003; Hobart et al., 2007).

## Measurement Properties and Considerations

Many guidance documents describe the criteria by which measurement tools should be evaluated (Finch et al., 2002; Revicki, 2007; Schoneveld et al., 2009; Baker et al., 2011) and funding agencies have published guidelines outlining the scientific requirements for patient-reported outcomes (PROs) and health-related quality of life (HR-QOL) measures (Scientific Advisory Committee of the Medical Outcome Trust (SAC MOT), 2002; United States Food and Drug Administration (USFDA) 2006). Despite their focus, these latter guidelines are pertinent to all measurement tools, including those of fine motor skills in people with tremor disorders.

A crucial first step in identifying and selecting an appropriate measurement tool is to define the purpose for which the measurement will be used. This helps clarify the reason for obtaining the measurement, identify the patient population being targeted, determine the setting in which the measurement tool will be used, and specify the aspect(s) of upper extremity fine motor skills that need(s) to be measured. Only when the measurement purpose has been clearly defined is it possible to properly evaluate and select the most appropriate measurement tool.

Table 1 presents key attributes that are used to describe the qualities and features of measurement tools (Baker et al., 2011; Finch et al., 2002; Scientific Advisory Committee of the Medical Outcome Trust (SAC MOT), 2002; United States Food and Drug Administration (USFDA), 2006). It includes a brief definition of each attribute and questions for consideration when selecting a tool to measure fine motor skills for people with tremor disorders. Table 1A presents attributes that apply to all measurement tools and includes several familiar terms, a few of which will be highlighted here. The *conceptual and measurement model* and *scaling assumptions* of a tool are closely related to *validity*. However, an explicit definition of the construct being measured is not always provided by the tool developers, or the definition describes the items of the tool rather than the construct it intends to measure. It is therefore important to ensure the measurement tool or one of its sub-scales focuses specifically on the construct of fine motor skills. *Responsiveness* is an important attribute when tracking disease progression or assessing the effectiveness of an intervention aimed at reducing tremor severity and improving hand function. For a tool to be responsive it must generate rigorous scientific measurements; unfortunately this is not always the case in measures of activity limitations (Hobart et al., 2007). When a measurement tool generates ordered scores, it may be assumed that increments in score are equivalent across the range of possible scores, i.e., that it is a linear measure. However, the nature of this relationship is often not known, which hampers the interpretation of scores. Modern psychometric methods such as Rasch analysis and Item Response Theory can improve a tool’s properties and render them rigorous scientific measurements (Hambleton and Swaminathan, 1985; Andrich, 1988); the use of such methods is still not widespread in clinical research.

**Table 1:** **Attributes of measurement tools**.

Attribute	Definition	Questions when selecting a measurement tool for fine motor skills in movement disorders involving tremor
**(A) ATTRIBUTES OF ALL MEASUREMENT TOOLS**		
Conceptual and measurement model	Rationale for and description of concepts and populations a measure is intended to assess, and in what populations	Does the tool evaluate fine motor skills *per se*? Was it developed for a particular patient population? Does it measure a broad construct, within which fine motor skills are merely a component? Does it purport to represent the progression of a specific disease? Or does it purport to represent impairment or disability more generally?
Scaling assumptions	Degree to which it is legitimate to sum scale or subscale scores, implying the sum reflects a common underlying construct	Are the items in the scale or subscale related to a common underlying construct relevant to fine motor skills?
Validity	Degree to which instrument tools measures what it purports to measure, including content, construct, and criterion validity	Do the tool’s scores have known relationships to any other measures of fine motor skills?
Reliability	Degree to which measure is free from random error, including test-retest and inter-rater reproducibility	Are scores consistent across raters and on separate occasions when patient status is thought to have remained stable?
Responsiveness	Ability to detect change over time that is clinically relevant	Does the tool have sufficient responsiveness to indicate when a clinically meaningful change in fine motor skills has occurred? Is the minimal clinically important difference known for the population being investigated?
Targeting	Extent to which items of a tool are acceptable for the population under investigation	Is the tool targeted to a specific movement disorder involving tremor or is it generic, potentially applicable to people of various conditions? Also, are the scores from subjects with tremor likely to cluster near the bottom (floor) or top (ceiling) of the possible range of scores on the measure? *See also scope of hand function, below*
Data completeness/quality	Degree to which all items of a tool can be obtained in each individual being evaluated	Is there a risk of missing data when the tool is used in subjects with movement disorders involving tremor?
Interpretability	Degree to which one can assign meaning to a tool’s quantitative scores	Is the relationship between measurement tool scores and the continuum of the construct being measured linear? Are there established norms for age and/or gender in healthy subjects, movement disorders involving tremor, or other diseases?
Burden: respondent and administrative	Time, effort, cost, personnel, or other demands required to complete the tool	Is it uncomfortable, frustrating, or embarrassing for subjects to answer the questions or do the tasks? What is the cost and portability of the instrument? Are there ongoing costs? How much experience is required by the evaluator to obtain valid and reliable scores in the population of interest?
**(B) ATTRIBUTES OF TOOLS TO MEASURE HAND FUNCTION**		
Scope of hand function	Extent to which tasks or items adequately capture the construct of fine motor skills	Are the conceptual model, scaling assumptions, and targeting of the tool appropriate for the selected aspect of hand movement function? Is the task (or set of tasks) appropriately comprehensive with respect to hand dexterity for the population in whom one intends to use the tool?
Handedness and bilateral tasks	Extent to which each hand is evaluated separately, and bilateral hand tasks are also evaluated	Because tremor can affect right and left hands differently, does the tool adequately capture each hand’s movement ability? If capturing natural function is important to the research or clinical question, does the tool include tasks that are normally done with two hands?
Relationship to learning or practice	Extent to which the performance of tasks or items is influenced by whether they are familiar and well-practiced	Are the tasks well-practiced for one hand, both hands, or neither? Have people learned compensatory strategies to perform tasks more quickly or smoothly? Is the familiarity and/or extent of compensation likely different between people?

Fine motor skills encompass a wide range of simple and complex tasks with different functional and physiological requirements (McPhee, 1987; Wiesendanger and Serrien, 2001; Jones and Lederman, 2006; Kus et al., 2011). Table 1B presents attributes that are specific to measurement tools focused on fine motor skills. First, the *scope of hand function* assessed by a measurement tool should be considered. Measurement tools can focus on a single-concept task, a more complex task, or a series of tasks ranging in complexity. Next, upper extremity tremor is often asymmetrical (Farkas et al., 2006; Louis, 2010) and in some cases related to *handedness* (Machowska-Majchrzak et al., 2011; van der Hoorn et al., 2012). A detailed evaluation of fine motor skills in tremor disorders requires selecting measurement tools that assess unilateral tasks in the dominant and non-dominant hands as well as *bilateral tasks* (Héroux et al., 2006). In other contexts, it may be more relevant to focus on a single task done with one hand. Finally, task familiarity can influence a performance and self-reported function. Performance of tasks usually improves with practice (Wulf et al., 2010; Taylor and Ivry, 2012) and individuals with tremor often develop compensatory strategies such as stabilizing their upper extremity on a firm surface to successfully accomplish them (Sanes et al., 1990; Pascual-Leone et al., 1993). These factors will impact tracking individuals over time or comparing between groups.

## Measures of Fine Motor Skills in Individuals with Tremor

Clinical measurement of people with tremor disorders has historically relied on disease-specific rating scales. In Table 2A, we show a selection of those tools that include components related to fine motor skills of the hands (reference citations for all measures discussed in this section can be found in Table 2.) All of these purport to measure impairment in functional tasks or specific movements that is likely to occur with a specific disease. With the exception of the PROs, the validity and reliability have generally only been proven with evaluators who are clinicians with some experience in movement disorders. These scales vary widely in the extent to which their items capture hand function and whether dominant hand principally, both hands separately or both hands simultaneously. Nevertheless, it must be recognized that the underlying construct for each tool appears to be “extent of disease impact” and not necessarily a representation of the scope of typical activities any person, regardless of disease, would perform with the hands. The tools designed for PD have a lower proportion of items related to hand function than those designed for ET or other movement disorders: logical because PD generally impairs movement of many body parts, and bradykinesia is often a greater source of impairment than tremor. Although the newer of these scales have had some clinimetric properties thoroughly examined, they have not focused on *interpretability* beyond the target disease population.

**Table 2:** **Selected measures related to hand dexterity**.

Name	Apparent construct	Burden	Focus on dexterity, handedness and bilaterality	Measurement	Interpretability
**(A) SCALES DESIGNED EXPLICITLY FOR MOVEMENT DISORDERS**					
MDS-UPDRS part II (ADL) (Goetz et al., 2008; Martinez-Martin et al., 2013)	Reduction in ADL abilities, mostly self-care, that are typically compromised in PD	PRO; if patient is not literate, a companion can assist with reading/ writing (contrast to original UPDRS part II which required experienced clinician to rate)	4–6 of 13 items rely on dexterity, depending how item is interpreted by each individual; handedness not considered, items performed with whatever hand is usual	Ordinal scale ratings	Designed only for PD. No population norms, other than expectation of “normal” rating on all items; Not systematically used in any other neurological or other medical condition. Sum of items interpretable as lesser or greater evidence of PD intruding on ADL
MDS-UPDRS part III (motor examination) (see citations above)	Impairment in simple movements that are typically compromised in PD	Experienced clinician must be available to rate the items, either by direct interaction or video observations	3 of 18 items, plus hand tremor and rigidity evaluated in other items; both hands evaluated but entirely separately; no two-handed tasks included	Ordinal scale ratings	As above, except that sum of items is interpretable as lesser or greater evidence of PD cardinal motor signs being present
FTM scale part B (Fahn et al., 1988)	Impairment in simple movements that are typically compromised in ET	Experienced clinician, *as above*	11 of 12 items; handwriting is only with usual hand; drawing tasks done with each hand separately; some others are two-handed tasks	Ordinal scale ratings	Designed only for tremor. No population norms, other than expectation of “normal” rating on all items; Not systematically used in any other neurological or other medical condition. Sum of items interpretable as lesser or greater evidence of tremor intruding on a variety of tasks
TETRAS – ADL subscale (Elble et al., 2012)	Reduction in ADL abilities, mostly self-care, that are typically compromised in ET	Experienced clinician, *as above*	8–12 of 12 items, depending on how some items are interpreted; some tasks would typically be done with dominant hand, but scoring for some items includes the possibility of using both hands for stability; others would naturally be two-handed tasks	Ordinal scale ratings	Designed only for tremor. No population norms, other than expectation of “normal” rating on all items; Not systematically used in any other neurological or other medical condition. Sum of items interpretable as lesser or greater evidence of tremor intruding on a variety of tasks
TETRAS – performance subscale (see citation above)	Impairment in simple movements that are typically compromised in ET	Experienced clinician, *as above*	3 of 9 items; one handwriting task; two other tasks done separately with each hand; no two-handed tasks included	Ordinal scale ratings	As above, except that sum of items is interpretable as lesser or greater evidence of tremor being visible in specific movement tasks
SPDDS (Biemans et al., 2001)	Self-perception of difficulty with a set of tasks, mostly self-care and household	PRO; if patient is not literate, a companion can assist with reading/ writing	13–16 of 24 items; most hand tasks typically are done with two hands, although one may be supportive rather than having fine motor control	Ordinal scale ratings	Designed only for PD. No population norms, other than expectation of “able to do alone without difficulty” rating on all items; Not systematically used in any other neurological or other medical condition. Sum of items interpretable as lesser or greater evidence of PD intruding on ADL, as perceived by the person him/herself
TDQ (Louis et al., 2000)	Self-perception of difficulty with a set of tasks, many self-care, tasks are likely familiar and commonly compromised by tremor	May be administered by interview or configured to be a PRO	31 of 36 items; most hand tasks typically are done with two hands, although one may be supportive rather than having fine motor control	Ordinal scale ratings	Designed only for hand tremor. No population norms, other than expectation of rating that “tremor does not affect the activity” on all items; Not systematically used in any other neurological or other medical condition. Sum of items interpretable as lesser or greater evidence of tremor intruding on ADL, as perceived by the person him/herself
**(B) SCALES AND TOOLS DESIGNED FOR HAND FUNCTION**					
ABILHAND (Penta et al., 1998)	Self-perception of manual ability in daily activities	PRO; if patient is not literate, a companion can assist with reading/ writing	23 of 23 items; most hand tasks typically are done with two hands, although one may be supportive rather than having fine motor control	Ordinal scale ratings	Designed for hand function in people with neurological or orthopedic disorders. Results in a person with tremor could be compared to those with stroke (Wang et al., 2011), CP (Arnould et al., 2004), MS (Barrett et al., 2012), systemic sclerosis, and neuromuscular disorders (Arnould et al., 2012)
Action Research Arm test (Lyle, 1981)	Grasp, grip, pinch, and gross movement of the upper limbs	Evaluator needs no clinical expertise, needs only know the tasks and scoring criteria. Specific but common items required: balls, blocks, etc.	All 19 items reflect hand function; 16 of 19 reflect fine movement of hands or fingers; both hands evaluated but entirely separately; no two-handed tasks included except one screening task (pouring) is two-handed	Ordinal scale ratings	Designed for hand function in people with neurological disorders. Results in a person with tremor could be compared to those with stroke (Platz et al., 2005; Lin et al., 2010; Baker et al., 2011; Chen et al., 2012; Connell and Tyson, 2012; Li et al., 2012)
TEMPA (Desrosiers et al., 1995b)	Time to complete common hand activities involving grasp, grip, transport, steady hold, and release; designed to capture age-related changes	Evaluator needs no clinical expertise, needs only know the tasks and use stopwatch. Specific frame and items required: e.g., spoon, key, etc.	Nine of nine items; four items are one-hand tasks, evaluated separately for each hand; five items are two-handed tasks, done according to individual preference	Time to complete tasks; ordinal scale also available if person cannot complete a task	Published norms for adults of various ages (Desrosiers et al., 1995b; Nedelec et al., 2011). Results could also be compared to those with stroke (Higgins et al., 2006; Platz et al., 2009), ABI (Moseley and Yap, 2003), MS (Feys et al., 2007), and tremor (Héroux et al., 2006)
Box and Block test (Mathiowetz et al., 1985a)	Visuomotor control, hand grasp, transport, and release, all at speed	Evaluator needs only know the task and use a stopwatch. Requires bins and 100 blocks (2.5 cm cubes)	One task: involves grasp, lift, transport, and release of blocks, performed one-handed, each hand evaluated separately	Count of blocks moved	Published norms for adults of various ages (Mathiowetz et al., 1985a; Desrosiers et al., 1994). Results could also be compared to those with stroke (Higgins et al., 2006; Lin et al., 2010; Connell and Tyson, 2012), MS (Goodkin et al., 1998; Platz et al., 2005; Paltamaa et al., 2008), ataxia (Corben et al., 2010), and tremor (Héroux et al., 2006)
Purdue pegboard test (Tiffin and Asher, 1948)	Visuomotor control, fingertip pinch, and release	Evaluator needs only know the task and use a stopwatch. Requires specific peg board and peg items	All tasks focus on control of hand in space and fingertip pinch and release; each hand is evaluated separately, then hands evaluated together in parallel, and also in two-handed assembly task	Count of pegs, or count of assembly items in final task	Published norms for adults of various ages (Desrosiers et al., 1995a; McCurry et al., 2001; Onder et al., 2002, 2005). Results could also be compared to those with PD (Adler et al., 2002; Proud and Morris, 2010; Postuma et al., 2012), tremor (Héroux et al., 2006; Sequeira et al., 2012), and MS (Gallus and Mathiowetz, 2003; Thickbroom et al., 2005)
Nine Hole Peg Test (Kellor et al., 1971)	Visuomotor control, fingertip pinch, and release	Evaluator needs only know the task and use a stopwatch. Requires specific pegboard and pegs	One task: involves grasp, lift, transport, placing, and release of pegs, performed one-handed, each hand evaluated separately	Time to complete task	Published norms for adults of various ages (Mathiowetz et al., 1985b; Oxford Grice et al., 2003). Results could also be compared to those with stroke (Higgins et al., 2006; Lin et al., 2010), PD (Haaxma et al., 2008, 2010; Earhart et al., 2011), MS (Goodkin et al., 1998; Feys et al., 2007; Kierkegaard et al., 2012; van Winsen et al., 2010), and ataxia (Corben et al., 2010)
**(C) SINGLE-CONCEPT TASKS**					
Finger tapping	Ability to tap one finger rapidly in specific time period, typically 10s, a component of psychomotor speed	Varies: may use specific-built device or use standard computer input equipment; alternatively, may use observation or video recording	Single task: tapping as fast as possible, typically only with index finger; each hand evaluated separately	Simplest is count of taps in 10 s; different tasks and/or calculations possible	Depending on task used, results can be compared to published norms (McCurry et al., 2001; Jimenez-Jimenez et al., 2011). Tasks have been used in many populations with neurological disorders, including stroke (Gebhardt et al., 2008; Calautti et al., 2010), PD (Adler et al., 2002; Quencer et al., 2007; Haaxma et al., 2008, 2010; Yokoe et al., 2009; Kim et al., 2011), and ET (Jimenez-Jimenez et al., 2010)
Spiral drawing	Ability to perform smooth circular movement required to draw or trace an Archimedes spiral	Digitizing tablet and stylus needed; expertise needed in signal conditioning and spectral analysis; alternatively may use simple paper version	Single task: drawing an Archimedes spiral with a stylus; possible to evaluate both hands, although rarely reported	Various, e.g.: peak spectral tremor velocity (mm/s)	Tasks have been used in people with Parkinson’s disease (Pullman, 1998; Saunders-Pullman et al., 2008), ET (Pullman, 1998; Haubenberger et al., 2011; Louis et al., 2012), MS (Feys et al., 2007), and Niemann-Pick disease (Hsu et al., 2009)
Precision grip and/or lift	Ability to perform precise gripping and/or lifting of a small object	Technology needed to obtain force and kinematic measurements; expertise needed in signal conditioning and spectral analysis	Single task: involves gripping and lifting a small object held between fingertips; possible to evaluate both hands, although rarely reported; bilateral grip tasks rarely reported	Various, e.g.: maximum grip force (N), maximum acceleration (mm/s^2^)	Measures from such tasks have been shown to be significantly different in controls and people with stroke (McDonnell et al., 2006), PD (Fellows and Noth, 2004; Benice et al., 2007), ET (Stani et al., 2010), Huntington’s disease (Rao et al., 2011), or MS (Reilmann et al., 2013)
Coin rotation	Ability to rotate a coin in one hand, typically using thumb, index and long fingers	Varies; may use live observation or videorecording; coin is typically a nickel (mass 5 g, diameter 21 mm)	Single task: rotating a coin in one hand; possible to evaluate both hands, although rarely reported	Count of 180° or 360° rotations of the coin in 10 s, or time to perform 20 rotations	Depending on the measure used, there may be published norms (Mendoza et al., 2009). Task has been used in people with left or right hemisphere damage (Mendoza et al., 2009) and PD (Quencer et al., 2007; Gebhardt et al., 2008; Foki et al., 2010; Lee et al., 2010; Vanbellingen et al., 2011) and MS (Kamm et al., 2012)

*MDS-UPDRS, Movement Disorder Society Unified Parkinson Disease Rating Scale; ADLs, activities of daily living; PD, Parkinson’s disease; FTM, Fahn-Tolosa-Marín scale; TETRAS, Tremor Research Group Essential Tremor Rating Assessment Scale; ET, essential tremor; SPDDS, Self-assessment Parkinson’s Disease Disability Scale; PRO, patient-reported outcome; TDQ, Tremor Disability Questionnaire; CP, cerebral palsy; MS, multiple sclerosis; ABI, acquired brain injury; TEMPA, Test Évaluant la performance des Membres supérieurs des Personnes Âgées*.

In Table 2B, we list a selection of tools designed to measure hand function in ways that are useful for, but not specific to, neurological disorders. The ABILHAND is the only tool in this list that is a PRO. Although initially developed to capture hand disability in people with rheumatoid arthritis, it has since been used in people with stroke, multiple sclerosis, and neuromuscular disorders. Moreover, it was developed with more attention to measurement theory and rigorous testing than most tools in Table 2. As a result, its items are the most representative set of hand function tasks among the tools listed. Nevertheless, as a PRO, it captures what a patient prefers to do with either hand rather than systematically evaluating both right and left hands. The Action Research Arm Test (ARAT), originally developed for both rheumatological and neurological disorders, is best known as a measure of upper limb function used in patients with stroke. It is generic and has one of the lowest levels of *burden* among the tools having a multi-item approach to evaluating fine motor skills. The other four tools listed in Table 2B – the Test Évaluant la performance des Membres supérieurs des Personnes Âgées (TEMPA), the Box and Block Test (BBT), and the two pegboard tasks – differ from the tools described above in two important ways. The first is that they are scored according to a physical value rather than an ordinal rating. The TEMPA and the Nine Hole Peg Test (NHPT) both have an outcome of time in seconds. The BBT and Purdue pegboard test both have an outcome of the number of objects moved in a set time period. The distinct advantage of tools with such outcomes is that intervals between scores are even and a ratio of two scores is valid: e.g., 20 s is twice as long as 10 s whereas an ordinal score of 4 would not necessarily represent twice as poor as a score of 2. As a result, the tools with timing or counting may be better at contrasting two people’s performances or tracking changes over time – i.e., they have better *responsiveness*. The second difference is that these tools all have published norms for adults of various ages. Tools with timing or counting outcomes are in contrast to ordinal scale systems in which individuals without disease are mostly presumed to be rated at the score indicating no evidence of disease or impairment (typically score 0). Tools with physical value outcomes thus assist clinicians and researchers to put an individual’s results in the context of normal human variability, including age-associated changes – i.e., they have better *interpretability* for some contexts. Among this list, ABILHAND, ARAT, BBT, and NHPT are all highly recommended tools for people with stroke and other neurological conditions (Lin et al., 2010; Baker et al., 2011; Connell and Tyson, 2012) based on *responsiveness*, *reliability*, and *validity*. The BBT and NHPT have already been used in ET (see Table 2); the ABILHAND and ARAT may also have value in people with tremor disorders.

In Table 2C, we show a selection of fine motor skill measures that focus on a single task and may use technology to capture movement features. Like the tools in Table 2B with time units or counted objects as an outcome, these approaches result in scores that are physical values and generally have greater *responsiveness* and *interpretability* than ordinal scales. In contrast to those from Table 2B, however, these approaches do not attempt to reflect fine motor skills in multiple ways. Rather, they rely on a single task as informative about general dexterity. Finger-tapping and spiral-drawing both have a long history. For both tasks, the *interpretability* is increased by the availability of comparison data for healthy controls and other neurological populations. Precision grip-and-lift tasks generally have a high technology *burden*, but the outcomes have shown important differences between healthy controls and people with stroke, PD, ET, or Huntington’s disease. Coin rotation is a relative newcomer in this category and its *interpretability* is growing as reports are published of its use in multiple populations. All four of these, and many other single-concept tasks, have the advantage of being simple ideas for a patient to understand and thus can be used in many populations. However, it is less plausible that single-concept tasks can validly represent the whole construct of fine motor skills (Hobart et al., 2007).

## Summary and Recommendations

Measurement tools need to measure the constructs they intend to measure. They also need to be clinically meaningful and interpretable. In people with tremor disorders, however, no single measure of fine motor dexterity possesses all the attributes that would make it optimal for all research and clinical situations and with all patient populations. Each tool has advantages and disadvantages, and the importance of each is context-dependent. We recommend that researchers and clinicians consider the questions in Table 1 and ask themselves which attributes are most important for the tool(s) to be used in any context or project, and whether a single tool or a set of tools is most appropriate.

Patient care and research funding guidelines increasingly require the use of scientifically sound measures that capture all relevant aspects of patient status. In contexts where it is relevant to measure fine motor dexterity separately in both hands using a task that is quickly accomplished and allows comparison across patient populations, tools like coin rotation, finger tapping or peg tests would be most appropriate. In other contexts, it may be more relevant to capture the patient’s perception of the dexterity limitations and be able to compare across patient populations, in which case the ABILHAND is the only one of our example tools that would suffice. In major research studies, when time, money, and other resources are invested to determine if a new treatment reduces hand tremor and improves dexterity, the measurements should reflect both these hypothesized benefits and a set of tools may be needed to capture a comprehensive picture of a treatment’s benefits. For dexterity, this could include simple measures like coin rotation and the BBT, more complex measures like peg tests and the ARAT or TEMPA, and a measure of patient’s perceived dexterity limitations such as the ABILHAND. If a choice exists, tools that generate interpretable, rigorous scientific measures should be favored.

Developing a new measurement tool is a large undertaking and requires considerable expertise and resources. Researchers are encouraged to consult with a health measurement specialist prior to starting and consider that it may be simpler to determine the measurement properties of an existing tool for a new application. In line with this idea, centers, and networks that are able to gather data from large cohorts of patients should consider investigating the attributes of existing tools, not only validity and reliability but also attributes like responsiveness and interpretability using modern psychometric methods such as Rasch analysis and Item Response Theory. At an individual level, clinicians and researchers can critically examine the tools used by their peers in the grants and manuscripts they review.

We hope that this mini-review serves to provide some guidance to clinicians and researchers who intend to measure fine motor skills in individuals with tremor disorders. Although we have attempted to provide current information, our selection of tools was intended to be illustrative rather than comprehensive. Moreover, even for the tools we selected, future research to re-examine these tools may show them to have different, perhaps better, attributes than we have concluded.

## Conflict of Interest Statement

The authors declare that the research was conducted in the absence of any commercial or financial relationships that could be construed as a potential conflict of interest.
